# Interfaces with Structure Dynamics of the Workhorses from Cells Revealed through Cross-Linking Mass Spectrometry (CLMS)

**DOI:** 10.3390/biom11030382

**Published:** 2021-03-04

**Authors:** Umesh Kalathiya, Monikaben Padariya, Jakub Faktor, Etienne Coyaud, Javier A. Alfaro, Robin Fahraeus, Ted R. Hupp, David R. Goodlett

**Affiliations:** 1 International Centre for Cancer Vaccine Science, University of Gdansk, ul. Kładki 24, 80-822 Gdansk, Poland; monikaben.padariya@ug.edu.pl (M.P.); jakub.faktor@ug.edu.pl (J.F.); javier.alfaro@proteogenomics.ca (J.A.A.); robin.fahraeus@inserm.fr (R.F.); ted.hupp@ed.ac.uk (T.R.H.); 2 Protéomique Réponse Inflammatoire Spectrométrie de Mass—PRISM, Inserm U1192, University Lille, CHU Lille, F-59000 Lille, France; etienne.coyaud@inserm.fr; 3 Institute of Genetics and Molecular Medicine, University of Edinburgh, Edinburgh, Scotland EH4 2XR, UK; 4 Department of Biochemistry & Microbiology, University of Victoria, Victoria, BC V8Z 7X8, Canada; 5 Genome BC Proteome Centre, University of Victoria, Victoria, BC V8Z 5N3, Canada

**Keywords:** cross-linking mass spectrometry, proteomics, chemical cross-linkers, CLMS, protein–protein, protein–DNA, protein–RNA interactions, structural biology

## Abstract

The fundamentals of how protein–protein/RNA/DNA interactions influence the structures and functions of the workhorses from the cells have been well documented in the 20th century. A diverse set of methods exist to determine such interactions between different components, particularly, the mass spectrometry (MS) methods, with its advanced instrumentation, has become a significant approach to analyze a diverse range of biomolecules, as well as bring insights to their biomolecular processes. This review highlights the principal role of chemistry in MS-based structural proteomics approaches, with a particular focus on the chemical cross-linking of protein–protein/DNA/RNA complexes. In addition, we discuss different methods to prepare the cross-linked samples for MS analysis and tools to identify cross-linked peptides. Cross-linking mass spectrometry (CLMS) holds promise to identify interaction sites in larger and more complex biological systems. The typical CLMS workflow allows for the measurement of the proximity in three-dimensional space of amino acids, identifying proteins in direct contact with DNA or RNA, and it provides information on the folds of proteins as well as their topology in the complexes. Principal CLMS applications, its notable successes, as well as common pipelines that bridge proteomics, molecular biology, structural systems biology, and interactomics are outlined.

## 1. Introduction

Decades of research into the cell biology, molecular biology, biochemistry, structural biology, and biophysics have produced a detailed understanding of individual DNA/RNA/protein molecules, and their interconnected networks. A great diversity of techniques has emerged for studying their structural interactions. However, even more complex, the structures of these workhorses from the cells are themselves dynamic, converting from one dominant form to another based on the proportions of particular proteoforms present for any given biomolecules. Accordingly, beyond the protein–protein/DNA/RNA interaction landscape, there is an entire universe to explore with respect to their structure and dynamics. One such high-throughput technique has emerged as a dominant player in understanding both interaction landscapes and their resulting protein/DNA/RNA structures, namely cross-linking mass spectrometry (CLMS). This review covers different methods that are available to study protein–protein/DNA/RNA interactions, and provides vital insight into CLMS, a collection of methods that are perfectly suited to achieve a better understanding of intra- or inter- molecular interactions.

**Figure 1:**
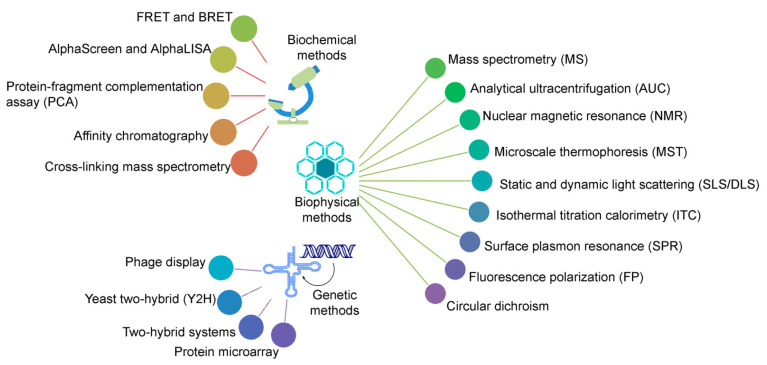
A spectrum of widely characterized experimental methods, based on biochemical, biophysical, or genetic principles. The listed methods define protein–protein interactions at various degrees of affinity as well as specificity [1,2,11,12,13,19,20,21,22,23,24,25,26,27,28,29,30,31,32,33,34,35,36,37,38,39,40]. The RNA and DNA diagrams, representing the genetic methods, were prepared using the BIOVIA draw (Dassault Systèmes, BIOVIA Corp., San Diego, CA, USA) tool.

Protein–protein interactions (PPIs) play a crucial role in all biological/biomolecular processes to understand the molecular mechanism of relevant protein molecules; hence, they are often termed the workhorses of cells. Over 80% of proteins do not function in isolation, but rather exist in interactions with one another to obtain stable or transitory complexes, as demanded by their observed function [1,2]. PPIs are considered to be an emerging class of drug target, since aberrant protein–protein interactions can participate in the pathogenesis of various human diseases, which, in turn, can contribute significant options for diagnostic as well as therapeutic targets. A large number of experimental methods have been developed to study PPIs, based on biophysical, biochemical, or genetic principles (as shown in Figure 1); and, each individual type has advantages as well as limitations regarding structural coverage of amino acid sequences, sensitivity, and specificity [1,3,4,5,6]. Methods can be chosen to emphasize different aspects of PPIs, such as identifying a protein binding partner(s), generating structural details of protein complexes, analyzing kinetic and thermodynamic constants of interactions, visualizing and quantifying PPIs in real time in living cells, and mapping small interactomes that refer to specific cellular pathways [1,3,4,5].

Mass spectrometry (MS) is one of the powerful approaches available that is useful in several areas beyond CLMS (e.g., hydrogen-deuterium exchange [7]), and it is particularly useful in combination with other techniques, providing steady progress for structural biology. Technical advances in mass spectrometry have made it possible to study protein–protein interactions from simple protein complexes to wide scale proteome experiments, which were not earlier accessible by traditional techniques [8,9,10]. Above all, mass spectrometry methods have democratized protein interaction analysis by making them accessible, relatively inexpensive, and high throughput. Having these advantages MS is becoming progressively popular in the structural biology stream for analyzing three dimensional (3D) structures and mapping their interactions with partner molecules. While emerging techniques, like cryoEM, can summarize large numbers of protein complexes, much of the resulting dynamics of the structure is missed and such gaps in structural data sets can be bridged by low-resolution methods, for example, chemical cross-linking (CL) [11,12,13]. The overall architecture of a protein complex can be obtained through electron microscopy (EM) [14], small-angle X-ray scattering (SAXS) [15], and ion-mobility (IM) MS [16], whereas the precise residues forming protein–protein interactions can be identified using hydrogen-deuterium exchange [17], chemical cross-linking [11,12,13], and chemical foot printing [18].

Fundamentally, the mass spectrometry methods simply measure a mass-to-charge ratio of an ion. Initially, analytes are ionized and then transferred into the gas phase prior to their separation according to mass-to-charge ratios in a mass analyzer. Subsequently, ions that emerge from the mass analyzer are recorded using a detector. The most common way to ionize a peptide or protein sample is electrospray ionization (ESI) [41]. However, a complementary ionization technique, known as matrix-assisted laser desorption/ionization (MALDI), is often used for its relative ease of use for novices. In a few studies analyzing intact protein complexes, MALDI mass spectrometry (MALDI-MS) has been used together with chemical cross-linking techniques [42,43,44]. Regardless of ionization methods, there are various types of mass analyzers accessible, including the following common ones: ion trap, time-of-flight (TOF), quadrupole, and orbitrap. Each of these mass analyzers may be configured in various ways with similar or different mass analyzers to form unique types of mass spectrometers [45]. 

Quantitative cross-linking mass spectrometry (QCLMS) approaches investigate protein structures as well as the dynamics of their interactions [46,47,48,49,50,51]. QCLMS is often performed using a unique cross-linker that introduces a corresponding mass shift after isotope labeling specific only to the cross-linked peptides [46,47,50,52,53], followed by quantitation of cross-links in MS1. However, the limited availability of isotope labeled cross-linkers restrains the implementation of this approach in QCLMS [54]. Stable Isotope Labeling by Amino Acids in Cell Culture (SILAC), which is a popular option is an alternative to the QCLMS approach, which relies on the metabolic incorporation of isotope labeled amino acids from culture media. Metabolically labeled samples are cross-linked and pooled; relative quantitation is performed from MS1 data using the characteristic mass shift introduced into peptides from the incorporation of isotope labeled amino acid/s [48]. SILAC enables a comparison of multiple samples per analysis (usually two) and it can also enable monitoring amino acid incorporation in a time course (pulsed SILAC), which is especially valuable for exploring dynamics in biological processes. Recently, iCLASPI (in vivo cross-linking assisted and stable isotope labeling by amino acids in cell culture (SILAC)-based protein identification), an approach combining SILAC and in vivo cross-linking, has been implemented to quantify native protein–protein interactions in HEK293T cells. iCLASPI has been successfully implemented to profile native protein–protein interactions involving core histones H3 and H4 in biological context [55]. Chavez et al. successfully implemented SILAC and cross-linking to investigate key protein–protein interactions, and then investigated the Hsp90 conformational changes upon treatment with 17-AAG Hsp90 N-terminal domain inhibitor [49].

Isobaric Tags for Relative and Absolute Quantitation/Tandem Mass Tag (iTRAQ/TMT) are chemical labeling, which are introduced to peptides after protease digestion, which allow relative protein quantitation via MS2 or MS3 encoded data. Nowadays, TMT/iTRAQ labeling enables comparison of up to 16 samples (valid for TMT) in a single MS analysis. Notably, the iTRAQ/TMT reporter ions used for quantitation are cleaved from labeled peptides during fragmentation by way of collision-induced dissociation allowing for the quantitation from fragment ion relative intensities. Yu et al. [56], implemented the TMT approach in a multiplexed comparison of protein complex dynamics and protein–protein interactions. Their QMIX (Quantitation of Multiplexed, Isobaric-labeled cross (X)-linked peptides) workflow with TMT labeling, achieves peptide quantitation from MS3 data that eliminates interference from ions that were observed in MS1 data along with isotope labeled cross-linkers or SILAC cross-linking [56]. Furthermore, the precise MS2 quantitation of protein cross-linking could also be achieved in a label-free manner utilizing a data-independent acquisition mode (DIA). The extraction of cross-linked peptide quantities from DIA data is usually performed utilizing a spectral library prepared particularly from investigated samples. Muller et al. developed a novel DIA-QCLMS approach utilizing photo activatable cross-linkers ensuring reliable quantitation of cross-linked proteins across a wide range of environmental changes, such as pH, temperature pressure, or concentration [51,52,57]. DIA approaches promise to be useful in future applications of quantitative cross-linking proteomics, due to their precision, reproducibility, and label-free manner.

Moreover, techniques, such as proximity-dependent biotin labeling (or BioID technique) in living cells, help to understand the plasticity of protein networks within heterogeneous cellular populations. In combination with nanopore technology, such an approach could help tackle pending biological questions, e.g., the identification of peptidyl-prolyl isomerases (PPIases) substrates. PPIases substrates preserve their primary structure/molecular mass, as well as the cis and trans isomers of the proline peptide bonds of the substrates interconversion by PPIases being the sole change. This subtle modification triggers important changes to the substrate’s fate, such as subcellular translocation, degradation, or rewiring of their protein–protein interaction networks. PPIase enzymes act as central molecular switches, as exemplified by the peptidyl-prolyl cis-trans isomerase NIMA-interacting 1 (Pin1) that has been extensively studied and showing its involvement in multiple diseases [58,59,60]. Herein, we propose that the CLMS techniques, though, which have not been previously carried out on such problems, could be merged with novel interactomics techniques (proximity-dependent labeling by BioID or an engineered biotin ligase by TurboID technique). Combining these two approaches may bring spatial resolution to CLMS at a sub organelle level, since the BioID radius is estimated ~10 nm and CLMS is aimed on the proteins in the neighborhood of a given bait.

While there has been some success in the proteome wide CLMS [61,62,63,64], in the case of both chemical cross-linking MS and the related techniques of native MS [65,66], a prior purification of single protein or protein complex is typically necessary, and this can be acquired through over expression with the purification of a recombinant version of particular system. When investigating the protein assemblies, individual components need to be purified to reconstitute the whole complex later in vitro and, alternatively, in vivo reconstitution can occur by co-expressing various subunits [67]. Because the reconstituted systems mostly benefit from large yields to aid the structural analysis and they are frequently done using bacteria (mostly *E. coli*) as the host system, functionally important post-translational modifications and interacting protein partner associations may be lost during this process. Thus, various biochemical approaches must be explored when beginning a new project in order to directly enable the isolation of endogenous protein complexes from cells or tissues [68].

Modern mass spectrometry that is coupled with the chemical cross-linking of juxtaposed amino acids can provide important structural information. Two main types of the cross-linking strategies involving either the activation of the cross-linking reagent by UV or chemical methods to enable cross-linking [11,12,13,69]. Chemical cross-linking is a classical approach for determining protein–protein interactions and is also one of the first approaches that has been used to map large complexes, for example, the ribosome [2]. Generally, the cross-linking techniques link two or more proteins present in a complex by covalent bonds and, as the name implies, via a molecule designed to bridge juxtaposed amino acids, i.e., to chemically cross-link residues. The chosen cross-linker is a chemical reagent that contains two or more reactive groups connected through a spacer or linker of various lengths [70]. By using this method, low-affinity protein–protein contacts, or some specific interactions, can be detected that are difficult to characterize by other methods (e.g., nuclear magnetic resonance (NMR), X-Ray, etc.). Moreover, the cross-linking techniques have also been applied to stabilize transient protein–protein interactions in a dynamic process both in vitro and in vivo [1,2]. However, there can be considerable weakness in these chemical methods that are related to the lack of spatial localization in a cell and a lack of control over activity. Thus, to evaluate PPIs as similarly as possible to the native conditions, photo-cross linking methods are valuable due to their ability to generate reactive species in situ instantaneously by irradiation with UV-light [11,12,13,69].

Cross-linking is always followed by other downstream methods to further analyze the cross-linked proteins, often using sodium dodecyl sulfate polyacrylamide gel electrophoresis (SDS-PAGE) to separate the cross-linked from non-cross-linked proteins, tandem affinity purification (TAP) or immunoaffinity chromatography for affinity-based purification of cross-linked products, and mass spectrometry methods for the interacting partner identification [71,72]. Additionally, SDS-PAGE analysis is very helpful in the early stages of analysis when empirically working out the correct ratio of cross-linker to protein complex. A limitation of using chemical cross-linking while using these methods is the high risk of detecting non-specific interactions. However, these limitations can be addressed using more than one cross-linker of differing activities or spanning various distances and by varying the ratio of reagent to protein complex. Non-specific interactions can result from proteins in close proximity that may not be functionally related. These suggest that while the CLMS technique is relatively straightforward to implement, the identification of relevant cross-linked proteins could be quite demanding due to the intracellular dynamic range of expression of proteins, which can range from one to one million copies low abundance of cross-linked species [1].

Innovative developments in the biological applications of MS led to the development of a large number of methods, and it has made it relatively simple to identify proteins alone or in complexes using CLMS technique. Large macromolecular complexes like ribosomes or exosomes, have been purified and analyzed directly using mass spectrometry [73,74,75,76]. Recently, chemical cross-linking combined with mass spectrometry based structural techniques have hit their stride allowing for various biologically relevant molecular machines to be successfully studied in the past few years using this combination [77]. The various workflows that have been developed to implement CLMS represent a vast toolkit that can help to provide novel insight into the structure and organization of proteins in order to define protein–protein interactions and probing PPI interfaces.

## 2. Concept and Perspectives of Cross-Linking Mass Spectrometry

**Figure 2:**
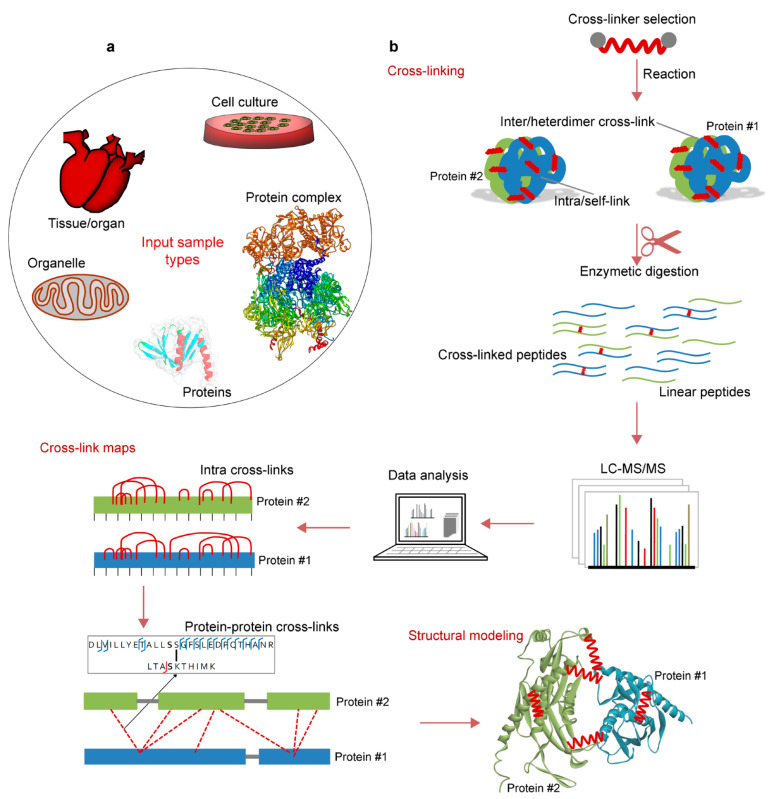
The generic workflow of cross-linking mass spectrometry (CLMS) experiments. (**a**) A wide range of samples (from purified proteins to intact tissues and organs) applicable to perform CLMS experiments [11,80]. (**b**) In a representative CLMS workflow, a selected linker is applied to the sample and the cross-linking reaction is carried out. Depending on the actual chemistry of interest, the reaction is stopped through chemical quenching or removal of the reagents. The proteins can be then digested in solution or gel to produce a mix of cross-linked and linear peptides. Prior to mass spectrometry analysis, the cross-linked peptides are often enriched by chromatographic methods, for example, the size exclusion chromatography, ion exchange chromatography, or purification through an affinity tag. Finally, the sample is subjected to LC-MS/MS (Liquid chromatography-tandem mass spectrometry) acquisition pipelines that have been developed to add the likelihood of selecting cross-linked peptide precursors for fragmentation. Using a variety of search software, two linked peptides can be identified from spectra and through the methods determining the false discovery rate, the list of matches can be filtered to the desired confidence. The cross-links can be also visualized by integrative modeling techniques. CLMS techniques convey the structural information in the form of distance restraints on single protein, protein complexes and allows to portray protein networks [11,80,81,82]. In this figure the structures of protein or peptides were prepared using BIOVIA Discovery Studio (Dassault Systèmes, BIOVIA Corp., San Diego, CA, USA) visualizer tool.

In the CLMS approach, chemical cross-linking reagents are used to join the components of interacting complexes, followed by LC-MS/MS (Liquid chromatography-tandem mass spectrometry) analysis that enables in vivo and in vitro methods to define the native PPIs of a protein complex, under optimal conditions. The visualization of the interacting regions allows distance maps within the protein complexes or within the protein to be created, e.g., low resolution three-dimensional maps of the interactions can be generated. In addition, to be valuable for defining protein–protein interactions, CLMS has emerged as a technique for interactomics and the structural biology of multi-protein complexes. For example, the CLMS based methods are now capable of capturing protein–protein interactions from their native environment, uncovering physical interaction contacts between them and, thus, providing the determination of both identity as well as connectivity of PPIs in cells (Figure 2) [78,79]. Some of the advantages of using the CLMS technique over traditional structural methods, like X-Ray diffraction, cryo-EM, or NMR include: limited amounts of starting material are required (i.e., typically only nanograms), no need for exceptionally pure protein(s), and much more rapid turnaround in the workflow allowing for more rapid hypothesis generation and testing. Those traditional approaches (*vide supra*) are limited by the particular proteins that can be easily expressed or crystalized, whereas the CLMS has an ability to examine the biologically relevant interactions that are close to the physiological state of an organism [77,78,79]. Moreover, PPIs must be very strong to survive the condition of extraction and purification required by the general sample preparation in traditional methods, and this makes it very challenging to analyze interactions in their native state. 

### 2.1. Associate Methods for CLMS Technique

**Figure 3:**
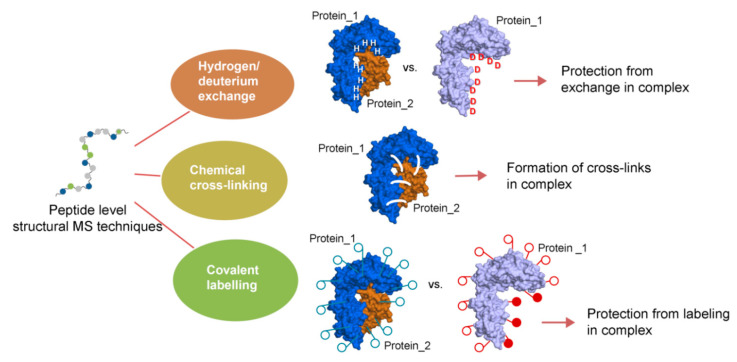
Three important mass spectrometry approaches used in the structural proteomics analysis of binary complexes. In the hydrogen/deuterium exchange, H_2_O is replaced by D_2_O and the resulting exchange associated with a mass increase can be detected by mass spectrometry (MS). Chemical cross-linking, comprises the covalent coupling of two reactive groups within a protein or between two different proteins, and then by introducing cross-links at the specific residues, spatial information at different levels can be obtained using MS. In covalent labeling, irreversible modifications are introduced at the reactive side chains and the solvent or surface exposed residues in proteins can be identified [23,72]. In this figure the protein or peptide structures were prepared using the BIOVIA Discovery Studio (Dassault Systèmes, BIOVIA Corp., San Diego, CA, USA) visualizer.

Three main chemical approaches that involve MS analysis on the peptide level are: (i) the exchange of labile hydrogen atoms with deuterium atoms in hydrogen/deuterium exchange (H/D exchange or HDX) methods, (ii) the covalent modification (i.e., painting or surface modifications) of amino acid residues (mostly the functional groups in side chains) in various covalent labeling workflows, and (iii) chemical cross-linking, in which two spatially proximate amino acid side chains are covalently coupled (Figure 3) [72]. Particularly, the CLMS methods are complementary to other MS techniques for structure analysis. As an example, the HDX-MS can provide information regarding the regions that may be analyzed by CLMS and it is frequently used to examine the conformational flexibility of protein complexes. Following this, CLMS can be used to obtain distances between interacting regions and fill in information that might be missed by traditional method, e.g., regions of NMR or X-Ray data that are poorly defined (Figure 3). When considering the fact that the CLMS workflows often involve simply injection peptide mixtures, as would be done for any shotgun proteomic experiment, there need not be any significant interruption to overall laboratory workflows apart from upstream use of specific cross-linking.

Despite the growing popularity of CLMS technique, there remain limitations that need to be overcome in order to make it more successful in defining the in vivo state of PPIs. Its main limitation is the small depth of interactome coverage [61]. To date, the ‘maximum number of cross-links identified in system-wide studies is ~10,000, howeverthis number is expected to grow as methods continue to improve. Specific challenges to overcome full interactome coverage are the high dynamic range of expression of proteins (from 1 to 10^6^ intracellularly) and diversity in their binding affinities. Additionally, these include the variety of amino acid residues that can be targeted by cross-linkers and the decreased solubility of cross-linked protein complexes that, in turn, causes problems during digestion and the ionization of large cross-linked products [11]. Addressable challenges include: (i) reducing sample complexity by liquid- and gas- phase methods, which, in turn, can increase detectable dynamic range, and thus, detect lower copy number proteins, (ii) targeting additional functional groups in proteins in an aqueous environment, and (iii) most CLMS methods target amine reactive cross-linkers that leads to a ‘dark’ interactome that is blind to PPIs, where the primary amine groups are absent or scarce in the contact region [11,83,84].

Identifying the potential regions of protein intra- and inter- molecular interactions is considered the main power of CLMS in part, because it can also provide direct information on a range of distance constraints that can be used to develop or enhance three-dimensional models of protein in complex structures [85,86]. However, rather than CLMS data being an end unto itself it is used as one of a set of analytical methods that, together, provide structural insights. In the case of CLMS, the main challenges are to achieve robust workflows that enable a comprehensive capture of dynamic biological systems interactions in their native environments in a routine manner [11]. Typically, the first step in any CLMS experiment is to define the chemical cross-linking reagents, which is based on known or expected amino acid sequence, be used, and then next to define the ratio of reagents to proteins. Inducing too many cross-links may make the complex undigestable, while too few may not provide useful information. This ratio is usually empirically defined by the following changes to the complex by SDS-PAGE. After this ratio is developed, the cross-linked proteins are digested to peptides using an appropriate enzyme. In some cases, an enrichment step may be integrated to isolate cross-linked peptides from the overwhelming number of non-cross-linked peptides in the sample that prohibits the detection of the lowest copy number cross-linked peptides. Finally, the peptides are introduced into an LC-MS/MS instrument for data collection, and specialized software packages designed for CLMS studies are then used for data interpretation (Figure 2) [72].

### 2.2. Chemical Cross-Linkers Structure and Chemistry

**Figure 4:**
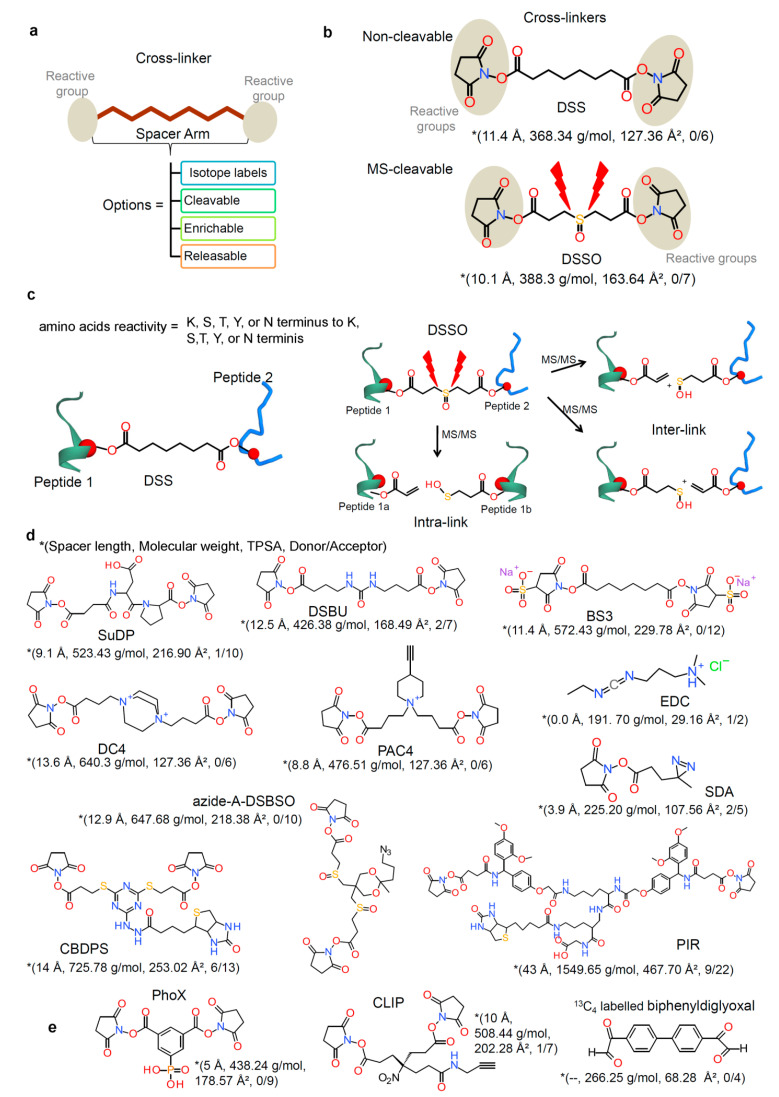
Cross-linking reagents anatomy, chemistry, and evolution of cross-linkers. (**a**) Cross-linking reagents comprise various reactive groups, spacer scaffolds with different lengths. Based on the applied experimental workflow, a variety of isotope labeled, cleavable, enrichable, releasable, and spacer groups are in use. A large number of cross-linkers establish their combinatorial complexity and further supports a larger number of future cross-linkers [87,88,89]. (**b**,**c**) Structures of the commonly used cross-linkers for analyzing protein–protein interactions involving non-cleavable (disuccinimidyl suberate, DSS) and MS-cleavable (disuccinimidyl sulfoxide, DSSO) cross-linkers [71,91]. Representation of the suggested fragmentation schemes (interlink as well as intralink cross-linked peptides) of DSS and DSSO cross-linked peptides. (**d**) In addition to DSS and DSSO, most commonly used cross-linkers are shown (SuDP, Disuccinimidylsuccinamyl aspartyl proline; DSBU, Disuccinimidyldibutyric urea; BS3, Bis(sulfosuccinimidyl) suberate; DC4, 1,4-Bis{4-[(2,5-dioxo-1-pyrrolidinyl) oxy]-4-oxobutyl}-1,4-diazoniabicyclo[2.2.2]octaneoctane; PAC4, 1,1-Bis{4-[(2,5-dioxopyrrolidin-1-yl)oxy]-4-oxobutyl}-4-ethynylpiperidin-1-ium; EDC, 1-Ethyl-3-(3-dimethylaminopropyl)carbodiimide; Azide-A-DSBSO, Azide-tagged, acid-cleavable disuccinimidyl bissulfoxide; SDA, succinimidyl 4,4’-azipentanoate; CBDPS, Cyanurbiotindipropionyl succinimide; and PIR, protein interaction reporter) [71,72,89,91,92,93,94,95,96,97,98,99,100]. (**e**) For the enrichment of cross-linked products/peptides a few specific cross-linkers areused such as:PhoX linker (non-cleavable but containing a phosphonic group) [101], CLIP cross-linker (introduces alkyne groups to cross-linked peptides) [102], and ^13^C labeled form of biphenyldiglyoxal (diglyoxal cross-linkers) [103,104]. For the cross-linkers on panel b, d, and e, different properties such as spacer length, molecular weight, topological polar surface are (TPSA), and number of hydrogen bond donor or acceptor atoms in the cross linker are described. TPSA and hydrogen bonds were computed using the Molecular Operating Environment (MOE; Chemical Computing Group Inc., Montreal, QC, Canada) package, and the protein/peptide/chemical structures were prepared using BIOVIA Discovery Studio visualizer and BIOVIA draw tools (DassaultSystèmes, BIOVIA Corp., San Diego, CA, USA).

A number of options for chemical cross-linking reagents with a wide variety of reactivities are available; they contain reactive ends to a variety of chemical specificity and these reagents are used to cross-link two regions within a protein (intramolecular) or between two different proteins (intermolecular cross-link; homo- or hetero- dimer). These intramolecular cross-links can stabilize tertiary or quaternary structures of a protein, and intermolecular cross-links stabilize the protein–protein complex (the main interest in the field of PPIs analysis) [87]. The growth in the use of protein CLMS has led to a wide variety of cross-linking reagents, with various distance constraints between functionally reactive groups. It may be required that more than one reagent each with different distance constraints could be used to aid construction of the three-dimensional protein structure or PPIs (Figure 4) [87,88].

In the simplest terms, chemical cross-linking reagents are comprised of two reactive functional groups that are separated by a spacer arm, which defines the distance between functional groups (Figure 4a). For an intramolecular (within a protein) cross-linking, a short spacer arm is more likely to be useful, whereas, for the intermolecular cross-linking studies, longer spacer arms can be more efficient. In addition, this difference is necessary to satisfy the steric constrain effects that guide the distance between potential reaction sites for cross-linking. The cross-linkers can be homobifunctional (having the same reactive functional groups at both ends of the spacer arm) or heterobifunctional (with different reactive groups at either end of the spacer arm). Homobifunctional cross-linkers are ideal for capturing a protein–protein interaction snapshot, while the use of heterobifunctional cross-linkers allow for two-step sequential conjugations that minimize undesirable polymerization or self-conjugation [87,88,89]. Overall, the chemical reactivity is the basis for choice of a given cross-linker. Different chemical properties that facilitate their use for specific applications and affect choice can be made by meeting specific criteria, including target functional groups, cell membrane permeability, and solubility. Cross-linking reagents are classified based on some general features, like chemical specificity (homo- or hetero- bifunctional structure), spacer arm length, water solubility, and cell membrane permeability (i.e., whether it is desired for the reagent to permeate cells or cross-link hydrophobic proteins within membranes), and instantly reactive or photoreactive groups [87,88,89].

The ideal cross-linkers for MS analysis with a focus on the different aspects of chemical structure and reactivity contain molecules with functional groups, by which at least two of the groups are reactive and capable of conjugation (Figure 4a). These types of cross-linkers are clearly the homobifunctional amine (lysine)-reactive N-hydroxysuccinimide (NHS) and sulfo-NHS esters (e.g., disuccinimidyl suberate, DSS; and, bis(sulfosuccinimidyl)suberate, BS3; Figure 4b and Table 1) [71,90]. Targeting the lysine residues is desirable due to their relatively high prevalence (~6% of all residues), their distribution across solvent-accessible protein surfaces, and the specificity of primary amine-targeting chemistries. Because it can be difficult to characterize the proteins with few or no lysine residues using these amine-reactive agents, other residues, like serine, threonines, and tyrosines, are also targeted for cross-linking [71,80]. The majority of recent studies have made use of non-cleavable cross-linkers and, thus, the DSS and BS3 have become the reagents of choice. The only difference between these cross-linkers is a sulfonic acid group that is incorporated into BS3 to improve water solubility, and to bridge a distance of 11.4 Å, which results in Cα-Cα distance of ~27 Å [71]. The tremendous success of DSS or BS3 reagents is due to their simplicity, reaction specificity, ease of use, reaction product stability, and lack of reaction by products. Their successful use has been demonstrated by integrative structural biology in purified complexes, in organelles, in cells, and labeled, as well as label-free quantitation for analyzing structural changes of protein [89]. Expanding further, MS-cleavable cross-linkers, such as disuccinimidyl sulfoxide (DSSO) and disuccinimidyldibutyric urea (DSBU), are also widely being used, since they provide an additional level of information in their tandem mass spectra that contain characteristic fragment ions generated during tandem MS experiments. The principal spacer lengths of cross-linkers in the community-wide CLMS study is in range of 10–12.5 Å, based on the most preferentially used cross-linkers, cleavable BS3 and DSS, as well as non-cleavable DSSO and DSBU (Figure 4 and Table 1) [71,91]. 

The spacer arm of cross-linkers can also act as a scaffold for functionalities that focus on the low abundance and complexity of analytes, as well as characteristics of cross-linking products that require analysis. Such functionalities involve MS-cleavable groups, isotope-coding, enrichment handles, and related capture, as well as release groups. Modular design approaches have been applied to cross-linker design and synthesis to generate multiple functional groups. For example, this concept is embodied by the popular protein interaction reporter (PIR) cross-linkers [89,92]. A class of PIR cross-linkers, containing MS-cleavable reagents that can also be enriched through a biotin label [93]. In 2006, a MS-cleavable cross-linker SuDP (Disuccinimidylsuccinamyl aspartyl proline) containing a labile aspartate-proline bond was presented [94]. In principle, the cross-link identification strategy that was presented for SuDP [95] formed a basis for the consecutive development of proteome-wide CL-MS workflows by using other MS-cleavable linkers. Various other MS-cleavable linkers, such as the DSBU [96], DSSO [83], CBDPS (Cyanurbiotindipropionyl succinimide) [97], and DC4 (1,4-Bis{4-[(2,5-dioxo-1-pyrrolidinyl) oxy]-4-oxobutyl}- 1,4-diazoniabicyclo[2.2.2]octane) [98], are revealed, as well as applied in different protein systems (Figure 4). As derivatives of DSSO, two different trifunctional cross-linkers were designed harboring azide (azide-A-DSBSO: azide-tagged, acid-cleavable disuccinimidyl bissulfoxide) or alkyne (alkyne-A-DSBSO) groups, which enable affinity purification approaches that are based on click-chemistry [99]. Likewise, the trifunctional PAC4 (1,1-Bis{4-[(2,5-dioxopyrrolidin-1-yl) oxy]-4-oxobutyl}-4-ethynylpiperidin-1-ium) linker was designed from DC4 having an alkyne group for an affinity enrichment of cross-links (Figure 4) [71,100]. The impressive array of chemistry applied for the enrichment of cross-linked products is provided by the PhoX linker (Figure 4e) [101]. The PhoX linkeris non-cleavable, but contains a phosphonic group, which allows for the cross-linked products to be enriched through routine methods, like titanium dioxide substrates, which are used for phosphopeptide enrichment by immobilized metal ion affinity chromatography (IMAC) [71].The purified cross-linker with the PyrR target protein complex was incubated to evaluate the diglyoxal-based (^13^C labeled biphenyldiglyoxal) cross-linkers (Figure 4e) and confirm their use to identify protein–protein interactions [103,104]. The work by AN Holding [103] described that these reagents (biphenyldiglyoxal; Figure 4e) were useful for recognizing the formation of arginine-selective cross-links in the human PyR complex.

### 2.3. Cross-Linked Sample Preparation for MS (Protein–Protein) Analysis

Protein cross-linking produces a challenging task for a mass spectrometrist, due to an introduction of multiple interconnected amino acid sequences into a mass spectrometer. Cross-linked proteins could be analyzed by either top-down or bottom-up mass spectrometry. Less frequent top-down mass spectrometry measures intact protein complexes; however, more common bottom-up mass spectrometry relies on simpler peptides that need to be generated prior MS measurement via proteolytic cleavage. Herein, we shed light on possible sample preparation pipelines used to turn a cross-linked protein sample into a clean peptide solution that is introducible into MS, and discussed the major factors impacting the detection of cross-linked peptides.

**Figure 5:**
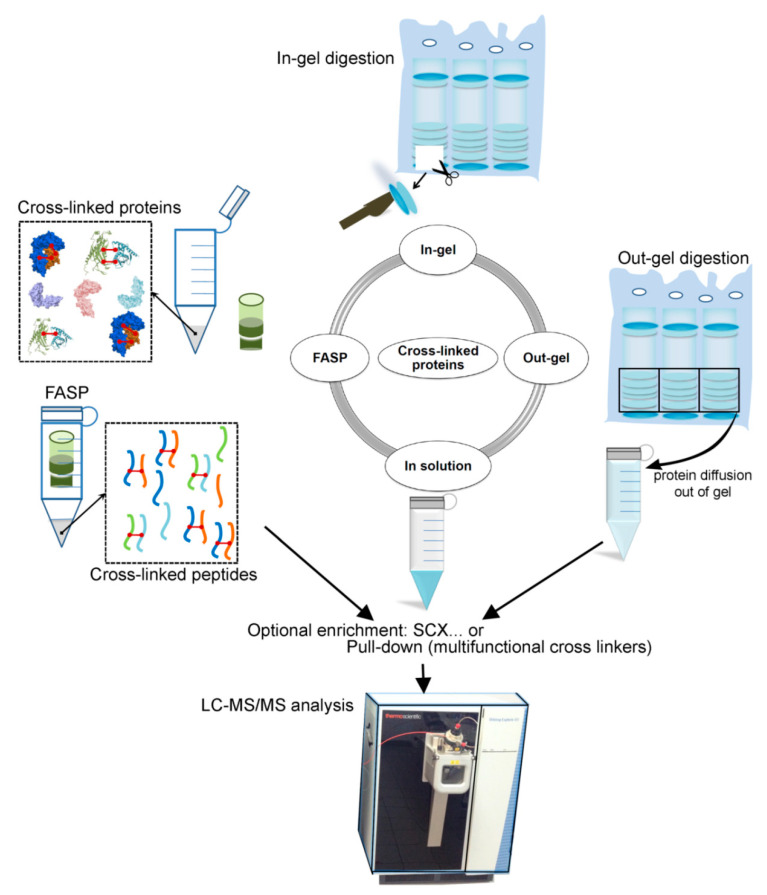
A workflow of determining protein structure, as well as the protein–protein interactions using the most frequent bottom-up cross-linking mass spectrometry. Extracted cross-linked proteins could be turned into peptides by several digestion protocols and proteases. Protein digestion protocol is chosen based upon up-stream steps taken to prepare cross-linked protein extract. FASP (filter aided sample preparation), ‘in-gel’, and ‘out-gel’ digestion protocols are used predominantly when a sample contains mass spectrometry incompatible substances, e.g., detergents or contaminants. However, ‘in-solution’ digestion might be used along with proteomic samples, which are already mass spectrometry compatible and contain predominantly cross-linked proteins of interest. Complexity of the cross-linked peptide samples might be reduced by separating cross-linked peptides from linear peptides after digestion. Difference in physico-chemical properties of cross-linked and linear peptides is often exploited to perform strong cation exchange or separation based on difference of peptide size. Alternatively, cross-linked peptides could be pulled down if a linker containing an affinity tag such as for example, biotin group has been used. Down-stream mass spectrometry analysis is frequently done in data-dependent acquisition mode (DDA). In the figure, representation of protein or peptide structures were prepared using BIOVIA Discovery Studio (Dassault Systèmes, BIOVIA Corp., San Diego, CA, USA) visualizer.

The peptide sample preparation process has a major impact on subsequent cross-linking experiments using mass spectrometry, because the investigation of protein interacting domains directly relies on peptides detectable by MS. The detection of peptides by MS is influenced by the chemicals used in sample preparation that may interfere with the eventual ability of any given peptide to be detected by ionization, and directly by the peptide’s chemical properties that make it more or less ionizable. The very first step after cross-linking is to ensure the cross-linked protein remains in solution, which can be a problem for some proteins that may have been marginally soluble prior to cross-linking. This decrease in the solubility may require the addition of chemicals, like detergents, to the buffer to improve solubility or simple dilution with lysis buffer. For all of these reasons, it is important to use mass spectrometry compatible buffers if downstream analysis does not or cannot include a buffer changing step. Rapid and effective cross-linked peptide detection also depends on the proteolytic digestion protocol. There are several cross-linked protein digestion protocols that have been reported (Figure 5).

Commonly, cross-linked proteins are electrophoretically pre-purified from organic contaminants and aggregates while using SDS gel electrophoresis, followed by the extrusion of peptides from the gel band after proteolysis in the same gel band. A study has been reported that focused on QCLMS [113], in order to investigate the protein structural conformations in solution used with in-gel digestion protocols (Figure 5). The digestion of photoactivated cross-linked proteins was performed after SDS pre-separation and concentration in polyacrylamide gel. Müller et al. claimed that they were able to detect 414 unique residue pairs, out of which 292 (70%) were quantifiable across triplicate analyses with a coefficient of variation (CV) of 10% [113]. In addition, several other studies suggested the use of ‘in-gel’ digestion in their cross-linking proteomics experiments (Figure 5) [114,115,116,117,118]. Nevertheless, the ‘in-gel’ digestion has the potential for the loss of cross-linked peptides, where their yield relative peptides without cross-linking is low. The main factors contributing to decreased cross-linked peptide recovery from a gel include poor peptide solubility, bulkiness due to the cross-link, and entrapment in gel pores due to peptide branched structure (Figure 5). Therefore, Petrotchenko et al. [119] created an ‘out-gel’ tryptic digestion procedure for chemical cross-linking studies with mass spectrometric detection. This out-gel digestion procedure is based on SDS-PAGE separation, followed by the passive diffusion of cross-linked proteins from the gel. Cross-linked protein digestion takes place outside the gel, which increases the probability that cross-linked peptides will be detectable (Figure 5). Petrotchenkoet al. included strong cation exchange (SCX) chromatography, followed by the zip-tip cleanup method on the C18 reversed-phase media to remove contaminants and salts from sample prior MS. Moreover, they showed that 93% of the cross-links have better or equal recovery while using ‘out-gel’ tryptic digestion, as compared to the standard ‘in-gel’ tryptic digestion [119].

The in-solution approach is another alternative that leads to improved cross-linked peptide recovery as compared to ‘in-gel’ digestion. It is important to ensure that the sample does not contain mass spectrometry incompatible detergents, and unwanted protein aggregates prior to ‘in-solution’ digestion (Figure 5). Mass spectrometry incompatible detergents and other organic contaminants will remain in the peptide solution, and they might interfere with peptide separation or cause ion suppression. Parfentev et al., in their study of the ‘n^2^’ problem of cross-linked peptide search, used ‘in-solution’ digestion to prepare a model of cross-linked proteomic data [120]. The ‘n^2^’ problem represents a challenge that consists in selecting any residue that is capable of cross linking, as a candidate to be cross-linked to any peptide considered in a specific experiment. Therefore, the ‘n^2^’ problem creates (n^2^ + n)/2 possible cross-links for ‘n’ number of peptides [121]. There have been many other reports relying on ‘in-solution’ digestion of cross-linked peptides [122,123,124,125,126]. A vital alternative to ‘in-gel’ and ‘in-solution’ digestion protocols, especially when the cross-linked sample contains detergents and contaminants, is ‘Filter Aided Sample Preparation’ (also termed FASP) method. The FASP method (Figure 5) employs a mass cut-off molecular filter allowing for the high recovery of cross-linked peptides after proteolytic digestion parallel with removal of organic contaminants. Rey et al. reported the use of eFASP protocol to digest membrane proteins cross-linked using trifunctional cross-linker, named ‘NNP9′, in the presence of a MS incompatible detergent [127]. In addition, they suggest that using eFASP followed by the enrichment of cross-linked peptides on monoavidin beads, leads to a drastic improvement in the number of identified cross-linked peptides, when compared to standard gel based digestion [127].

The protease or multiple proteases used to digest the cross-linked protein must be carefully chosen along with a digestion protocol, as this presents yet another factor that could substantially enhance or diminish the result of the experiment. This is in part due to limitations in mass spectrometry, and the typical use of acidic solutions to detect basic peptides by MS. Specifically, peptide length and physicochemical characteristics influence MS detectability as does the co-eluting non-peptide matrix of chemicals used for sample preparation. Recently, it has been shown that the detectability of large tryptic peptides could be enhanced by including stepwise multi protease digestion. Mendes et al. reasoned that sequential digestion could offer an access to the sequence space that otherwise would remain unseen, therefore they employed additional proteases such as AspN, LysC, and chymotrypsin. Multiple protease digestion could reveal mechanistically important protein regions that would not be detectable with tryptic digestion alone [110].

Cross-linked peptides represent only a relatively small portion of the peptide pool in any given cross-linked protein sample after proteolysis. Therefore, sometimes cross-linked peptide enrichment or peptide pre-fractionation steps are included. Ion exchange chromatography represents a potent tool to separate linear from cross-linked peptides, as cross-linked peptides have a higher isoelectric point and are naturally more likely to have higher charge states. Fritzsche et al. demonstrate the benefit of pre-separation after cross-linking by comparing the number of identified cross-linked peptides with and without prefractionation step. Their data clearly show the benefit of introducing SCX, which is reflected in a rapidly increased identification of interpeptidal cross-linking products and overall gain in structural information [128]. The ChaFRADIC (charge-based fractional diagonal chromatography) protocol has been developed to enhance SCX separation of linear from cross-linked peptides. In principle, it relies on blocking free primary amines with dimethyl chemistry prior to proteolysis. Following this reaction, cross-linked proteins are digested with trypsin and resulting peptides are separated by SCX into fractions with theoretical charge of +1, +2, +3, or +4. Subsequently, the peptides are trideutero-acetylated to block newly created N-termini. Then SCX is performed again and the net charge of internal peptides will be reduced by one, while net charge of N-terminal peptides will not be changed as they have already been dimethylated. Internal peptides shift to earlier fractions while N-terminal peptides elute the same as before [129]. Introducing SCX pre-separation step is popular in cross-linked proteomics as it is well documented in several studies (Figure 5) [119,128,129,130]. Nevertheless, SCX fractions require additional desalting steps prior mass spectrometry analysis which might lead to extensive peptide losses.

Alternatively, the cross-linked proteins might be enriched by pull-down via a biotin group or various other groups introduced during cross-linking reaction. Chowdhury et al. developed a novel multifunctional CLIP cross-linker (Figure 4e) that introduces alkyne groups to cross-linked peptides. This group could be exploited to enrich cross-linked peptides via alkyno-azide chemistry after the reaction [102]. Tan et al. developed trifunctional linker cross-linking free amino groups of interacting proteins. Their cross-linker contained a biotin tag for cross-linked peptide purification. Interestingly, the cross-linker also possessed a chemical cleavage site to detach biotin tags after purification. As needed a spacer arm can be included to introduce isotope-labels for quantitative purposes [131]. However, affinity purification of cross-linked products enriches the sample for peptides that reacted with cross-linking reagent, but it does not distinguish between linear and cross-linked peptides. Therefore, exploiting the size difference between linear and branched cross-linked peptides is another option of how to deal with low abundance of cross-linked peptides. Several studies employed a SEC (size exclusion chromatography) step to pre-separate emerging peptides based on their size [132,133]. Leitner et al. assembled a cross-linking workflow including SEC chromatography separating true cross-linked proteins from all kinds of linear proteins that does not contribute to a description of protein complex. Moreover, they demonstrate that the identification of cross-linked proteins increases 3-fold upon introduction of SEC [133]. Adopted workflow was then applied to enhance study of cross-linked protein complexes of human protein phosphatase 2A (PP2A) [134].

Identification of protein cross-links depends on additional factors such as the cross-linked peptide ionization efficiency, mobile phase composition, electrospray setting, mass analyzer, acquisition method, and data analysis. The cross-linked peptides could be eluted with other peptides (non cross-linked) or even with the contaminants, and therefore they will compete for a given number of protons or charges in the electrospray source. Peptides with better ionizability will tend to charge more and peptides with low ionization efficiency will be suppressed resulting in no or low signal. Moreover, gold standard DDA (data dependent acquisition) methods can detect only a limited set of charged precursor peptides at a given time. The DDA methods pick ions for tandem MS in stepwise fashion from the most abundant ion at any given time, which naturally excludes lower abundance ions. It is not uncommon that more peptides elute at the same time than can be selected for tandem MS, which could lead to complete exclusion of low intensity precursors ions, some of them may be cross-linked peptides [135,136,137,138]. Successful cross-linked peptide detection is only the very first step of a cross-linked experiment and always includes finely tuned downstream data analysis, which is another important step in the overall pipeline to identify juxtaposed amino acids.

### 2.4. Cross-Linking by UV for Protein–DNAInteractions

Protein-DNA interactions, fundamental to the functionality and stability of the genome, control essential cellular processes like replication, transcription, repair, and recombination. To understand the DNA-dependent processes, mapping of such protein–DNA interactions as well as identification of specific sites of interaction are required [139]. Many of the previously described CLMS workflows can be adopted to obtain such insights as has been recently demonstrated by Stützer et al. [140], who performed mass spectrometric identification of proteins interacting directly with DNA in reconstituted and native chromatin after cross-linking by UV light. Analysis of contact interface at amino acid level was possible by this approach, and they also described the possible means to distinguish the protein–DNA and protein–RNA interactions by performing a single experiment [140]. Chromatin is one of the most prominent protein–DNA complexes of a eukaryotic cell and in this, a core of eight histone proteins (2xH2A, 2xH2B, 2xH3, and 2xH4) associate with DNA in a repetitive manner to facilitate structural and functional organization of the genome [139]. Several studies described that RNA binding sites in proteins can be detected efficiently by using UV cross-linking combined with mass spectrometry [141,142]. Similarly for the cross-linking of protein and DNA components, a well established protein–DNA cross-links are induced in vivo after the exposure of cells to UV light, ionizing radiation or alkylating agents, in order that lead to bulky DNA lesions [140,143]. UV irradiation of DNA triggers a cellular cascade called DNA damage response, comprising a multitude of proteins and likewise, this UV irradiation has been successfully applied to cross-link single- and double- stranded DNA to proteins for analyzing chromatin dynamics [144]. Accordingly, the UV cross-linking with mass spectrometry could be beneficial to explore structural and functional relations in protein–DNA systems [140].

**Figure 6:**
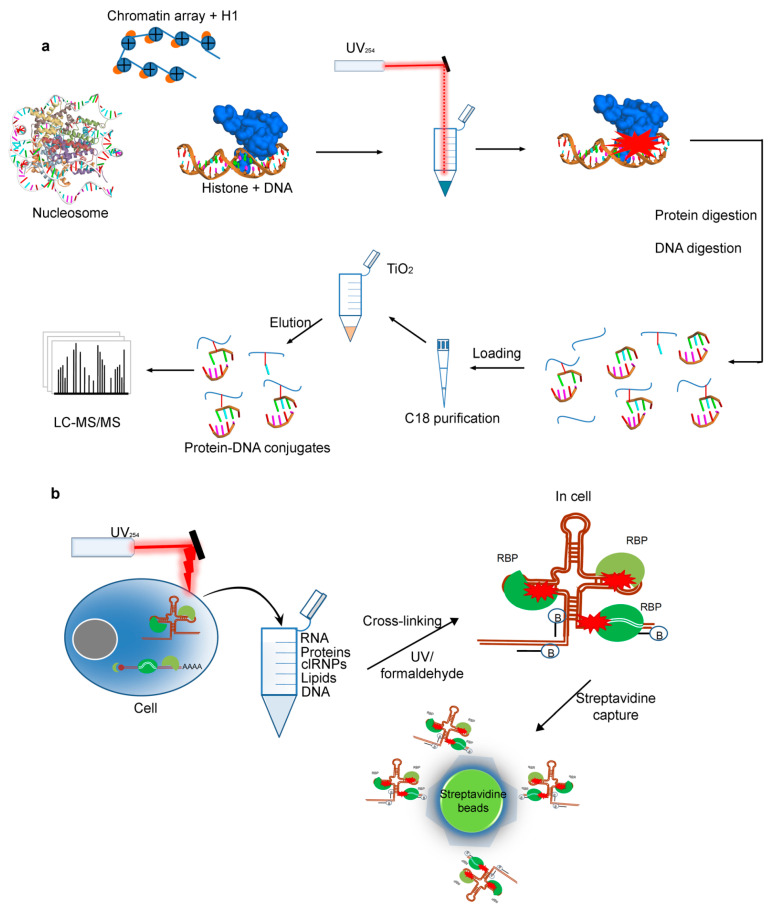
Schematic outline of the CLMS technique, to probe protein- DNA /RNA interactions. (**a**) Workflow for mass spectrometry analysis of UV-induced cross-links in histone-DNA complexes, nucleosomes, and chromatin arrays [140,159]. (**b**) Cross-linking based methods use either UV (RAP, RNA affinity purification; PAIR, peptide-nucleic acid assisted identification of RNA-binding proteins; MS2-BioTRAP, MS2 in vivo biotin-tagged RAP; and TRIP, tandem RNA isolation procedure) or formaldehyde cross-linking (CHART, capture hybridization analysis of RNA targets; ChIRP, Chromatin Isolation by RNA Purification) to capture RNA–protein interactions. Biotinylated oligonucleotide probes are hybridized to the RNA, and then the RNA and cross-linked proteins are purified for downstream analysis (RBP, RNA-binding protein; clRNPs, cross-linked RNPs) [149,160]. Representations of the protein or peptide structures in the figure, were prepared using BIOVIA Discovery Studio (Dassault Systèmes, BIOVIA Corp., San Diego, CA, USA) visualizer.

Recently, Stützer et al. adapted the established protein–RNA CLMS workflow for detecting protein–DNA cross-links [140]. In their initial experiments they showed that UV irradiation at 254 nm efficiently cross-link histone proteins to double stranded DNA. Consequently, they set out a protein–DNA cross-linking workflow that can be used for simple protein–DNA complexes, for example; oligonucleosomes, chromatin binding factors, and also complex systems like cell nuclei. In this workflow [140], first the linker histone-DNA complexes along with single nucleosome and 12 mer oligonucleosomal (chromatin) arrays containing Xenopus laevis core histones [145] were prepared. After irradiation, protein and DNA complexes were hydrolyzed with DNA nuclease and trypsin to generate peptides, oligonucleotides and cross-linked peptide-DNA oligonucleotide conjugates acceptable to MS analysis. Oligonucleotides without cross-links were excluded by C18 reversed phase chromatography and finally, peptide-DNA conjugates were enriched by using TiO2 affinity chromatography (Figure 6a) [141,146]. Purified peptide-DNA oligonucleotide conjugates were then analyzed through LC-MS/MS, and resulted MS data were analyzed using the RNP^xl^ computational workflow [141,147] in the OpenMS (https://www.openms.de (accessed on 23 February 2021)) software network (Figure 6a) [140].

Following the successful application of the workflow and encouraging results obtained in case of UV cross-linking of DNA-binding proteins in native chromatin, Stützer et al. [140], eventually applied it for analyzing protein–DNA interactions in more complex samples. Towards these ends, intact nuclei were isolated from HeLa cells and subjected to UV irradiation. Chromatin was isolated from the cross-linked nuclei by formaldehyde induced the cross-linking method based on chromatin-precipitation [148]. The isolated UV-irradiated chromatin fraction was digested while using RNase, DNase, and trypsin. The resulting mixture containing peptides, cross-linked species and oligonucleotides was then further processed (Figure 6a) [140]. In these initial in nucleo cross-linking experiments, Stützer et al. [140] hardly observed histone cross-links, and they referred it to the used purification strategy (TiO2-based enrichment without any further steps to remove the multitude of phosphorylated peptides or cross-links (peptide-RNA) that are present in UV cross-linked and digested nuclei). The co-enriched peptides block mass spectrometry detection as well as the sequencing of DNA cross-linked peptides. In order to overcome this obstacle, Stützer et al. [140] applied a more sophisticated enrichment strategy, in which they combined a size-exclusion chromatography (SEC) step along with chromatin-isolation and final TiO2 affinity enrichment. By removing most RNAs by RNase digestion and trypsinizing protein and maintaining the DNA intact, the larger-sized DNA-peptide cross-links were successfully separated from linear (phosphorylated) peptides as well as from the vast majority of peptide-RNA oligonucleotide cross-links (Figure 6a).

### 2.5. Protein–RNA Interactions Identified by Cross-Linking MS Technique

Non-coding RNA sequences involving long non-coding RNAs, small nucleolar RNAs, and untranslated mRNA regions make direct interactions with proteins to achieve their various functions. Recent efforts have categorized the methods for studying RNA–protein interactions in two different approaches, one that identifies proteins that are bound to RNA of interest (RNA-centric) and other that characterizes RNAs bound to a protein of interest (protein-centric). Herein, we review different methods for studying protein–RNA interactions, while focusing on the cross-linking MS technique (Figure 6b) [149].

#### 2.5.1. The RNA-Centric Cross-Linking

The changing patterns of RNA–protein interactions is critical for cellular functions, which are then regenerated based on the subcellular localization and environment stimuli [150]. Characterizing such protein–RNA interactions is challenging, as they are dynamic and transient. Generally, RNA-centric methods can be in vitro or in vivo, from which in vitro approaches are useful for analyzing RNA and protein molecules outside the context of a cell, while in vivo methods are useful to investigate such interactions within the cellular environment [149].

Cross-linking methods can be used to identify protein–RNA interactions in vivo. To do so, the RNA is purified under denaturing conditions to remove non-covalent interactions and only cross-linked components are subsequently extracted for identification. The formaldehyde, a small and bifunctional cross-linker, can easily permeate the cells and cross-links macromolecules within 2 Å, involving the protein–RNA complexes, by creating a reversible covalent linkage [151]. Methods that use formaldehyde to cross-link RNA to proteins are referred to as Chromatin Isolation by RNA Purification (ChIRP) [152] and capture hybridization analysis of RNA targets (CHART) (Figure 6b) [153]. Additionally, the UV light is a zero-distance cross-linker method and, thus, it cross-links protein to nucleic acid at a zero distance or in direct contact and in irreversible covalent bonds. Despite that the UV light is considered to be a more specific cross-linker, the efficiency of UV cross-linking is lower, it has slight uridine preference, and double-stranded RNA is known to be poorly cross-linked [154]. In vivo methods that use UV cross-linking involve RNA affinity purification (RAP) [155], peptide-nucleic acid assisted identification of RNA-binding proteins (PAIR) [156], MS2 in vivo biotin-tagged RAP (MS2-BioTRAP) [157], and tandem RNA isolation procedure (TRIP) [158]. Although all of these methods use a UV cross-linking approach, they have a different experimental setup.

The strength of the RNA–protein interaction should be mainly considered while choosing the cross-linking approach. RNA–protein dissociation constants vary widely, and such experimentally measured constants can be in the range of high nanomolar to picomolar concentrations. Generally, the weaker RNA–protein interactions are less likely to be captured by UV cross-linking as contrary to formaldehyde cross-linking. In addition, the UV cross-linking efficiency varies according to amino acid chemistry, whereas, in the formaldehyde cross-linking, nucleophilic lysine residues are strongly preferred and cross-linked (Figure 6b) [149]. Hence, more cells can be required to capture RNA–protein interactions with UV light cross-linking as compared to that of the formaldehyde method [149].

In the proteomic analysis, most of the RNA-centric methods follow quantitative mass spectrometry workflows to detect proteins that are bound to RNA. These MS approaches can be either label-free or incorporate the use of chemical labels for detection or simply quantitation. Labeling methods can use stable isotope labels or chemical tagging of proteins in samples (can be termed as controls) and, thus, the enrichment scores can be obtained by the ratio of labeled peptides and true binding partners can be identified [161]. Labeling MS techniques, like SILAC and iTRAQ, are especially effective with formaldehyde cross-linking. However, they are more expensive and desire greater technical expertise in MS data analysis. The main challenge of label-free MS is to distinguish true binding partners from non-specific proteins [162]. Analytical tools, for instance, SAINT (significance analysis of interactome score), can be useful for spectral count data from non-quantitative MS in order to effectively score the probability of a protein–RNA interaction in this case. Some of the methods require the purification of cellular RNA (e.g., CHART, RAP), validating the isolated RNA by sequencing will assure that proteomic analysis has certainly found interactions with RNA [149].

#### 2.5.2. The Protein-Centric Cross-Linking

The majority of studies identify RNAs that are bound to a protein are performed by purifying the protein of interest. The most common way to achieve this is by making use of the long-known fact that the protein will chemically cross-link to the nucleic acid in vivo when hit by UV light at ~254 nm [163]. This UV induced cross-linking played a key role in identifying RNA binding proteins, and nearly all amino acids cross-links excluding the residues: aspartic acid, glutamic acid, asparagine, and glutamine [141]. Techniques in which the UV cross-linking is followed by the protein purification and bound RNAs can be identified are widely termed cross-linking immunoprecipitation (CLIP) methods [164], along with those using the high-throughput sequencing (HTS) forming the CLIP-seq family of methods [165]. Approaches using a similar protocol, but with an alternative cross-linker, are also specified as CLIP methods. In some cases, where the indirect interactions are not bearable, alternative cross-linking reagents can be effective, for example, the PAR-CLIP (photoactivatable-ribonucleoside-enhanced cross-linking and immunoprecipitation) [166]. PAR-CLIP uses 4-thiouridine and/or 5-thioguanine as a nucleotide analog, and it is beneficial, especially where the UV light is not penetrating deep enough into the sample. However, the results have been mostly similar to those with the regular cross-linking technique [149].

Lately, the formaldehyde has been used as a cross-linking reagent for CLIP method on a double-stranded RNA-binding protein, which are, in most cases, thought to UV cross-link poorly [166]. Methylene blue was used to cross-link double stranded RNA to RNA binding proteins [167,168]; however, this method has not been broadly used in CLIP so far. Many compounds that are known to cross-link RNA to protein, like Dithiothreitol [169], 2-iminothiolane [170], and diepoxybutane [171], are not considered to bereagents for CLIP methods and, likewise, many other cross-linking compounds remained uncharacterized. Despite the use of either standard UV cross-linking or alternative methods, approaches that are based on protein purification for protein-centric RNA studies establish a backbone of the field and, among these, the leading methods are the rapidly expanding array of CLIP [149].

Furthermore, to develop the mechanisms for analyzing protein–RNA interactions is one of the possible, but not really touched, potential of cross-linking reactions. In order to identify such interactions, the use of diglyoxal compounds has been described in analyzing the ribosome organization [103,172]. These cross-linking reagents are determined to be applicable for studying protein structure with the identification of arginine-arginine cross-links [173] in proteins, as well as also useful in analyzing the nucleic acid structure [103,174]. AN Holding has described the successful use of diglyoxal cross-linkers [103,174], by developing a ^13^C labeled form of biphenyldiglyoxal (Figure 4e) from the friedel-crafts acylation reaction between biphenyl and ^13^C_2_-acetyl chloride, and that is then followed by oxidation of the terminal carbons using HBr/DMSO oxidation.

### 2.6. Pairing the CLMS Methodologies with Molecular Dynamics Simulations

Over the years, there has been a collection of biological data recorded for the purpose of building virtual biological models that can also be used for molecular dynamics simulation (MDS). Based on a general model of the physics (or biophysics), the MD simulation predicts the conformational dynamics or time course movements (at femtosecond resolution) of every atom from a biomolecule (e.g., protein, RNA, DNA, etc.) that is used to assist understanding of important biomolecular processes [175,176,177,178]. These simulation methodologies are often applied alongside several experimental techniques, for example, NMR [179,180]. The NMR technique can efficiently resolve secondary structure; however, a pairing between NMR cross-coefficients and MDS under NMR restraints/constraints enables the construction of self-consistent complete protein tertiary structures, which could even resemble physiological tertiary structures. These unique features of the deterministic MD simulation technique suggest that the MS-based approaches, such as the chemical-cross linking in combination with MDS, could play an important role in structural biology, and bring insights for several biomolecular processes. The CLMS methodologies provide inter-residue distances that can be integrated into the molecular modeling and simulation techniques (especially coarse grained MD) to achieve physiologically realistic quaternary (PPI) structures, which can be difficult to resolve by techniques, such as X-ray crystallography or cryo-EM.

Currently, significant progress is being made by pairing the cross-linking mass spectrometry with MDS in order to explore a wide range of biological questions concerning protein motions, interactions, and their assemblies. For example, Brodie et al. presented an integrative structural biology approach, in which short-distance cross-linking constraints are incorporated into rapid discrete molecular dynamics (DMD) simulations [181]. They provided a workflow on proteins with well-defined structures, and they have also validated the predicted structural models with other experimental structural proteomics approaches, namely: hydrogen-deuterium exchange, chemical surface modification, and long-distance cross-linking. Three main steps of their workflow involve: (i) the acquisition of short-distance cross-linking data, (ii) performance of DMD simulations that are guided by these cross-linking constraints, and (iii) validation of the obtained structures with additional structural proteomics methods [181,182]. Moreover, despite the in-depth information derived from the CLMS techniques, the cross-linking experiments can occasionally generate inconsistent data due to the fluctuations in solution structures of protein [181,183] and, thus, the inclusion of cross-linking constraints will define a structural ensemble instead of a single protein structure. Therefore, one must consider this while selecting the best-fit models from computationally produced ensembles of conformations [181,184], as well as while directly integrating distance constraints into an energy-based simulation process [181,185].

## 3. Conclusions and Outlook

Rapidly growing technologies to map out interactions between protein–protein /RNA/DNA are critically important since the biological function of these molecules is extremely influenced by their structures, complex formation, locations and regulatory networks. Herein, we reviewed a suite of methods that are valuable for detecting such interactions between biomolecules of the cell. Special emphasis was on the combination of cross-linking with native mass spectrometry technique to yield mutual benefits while characterizing protein–protein, protein–RNA, or protein–DNA interactions, though, with a slightly modified approach. The CLMS strategies allow for capturing and identifying not just stable, but also transient, dynamic, and weakly bonding molecules and, thus, it emerged as the most striking example of multidisciplinary success among hybrid or integrative structural biology methods. In addition, CLMS provides inter-residue distances that can be integrated into the molecular modeling and MD simulation techniques, in order to achieve physiologically realistic quaternary (PPI) structures. Different means to combine the knowledge of chemistry with MS to analyze essential biological systems of interest were scrutinized. Several different cross-linkers are accessible with varied chemistries for the CLMS techniques and, therefore, one should consider the protein sequence of interest to determine what sort of combination of cross-linker and cleavage enzyme yield positive results. To start with one of the most commonly used cross-linker types, like amine reactive cross-linking (NH2-NH2: BS3 and DSS, spacer length 11.4 Å; and BS2G, spacer length 7.7 Å) and carboxyl-amine cross-linking (COOH-NH2: EDC or sulfo-NHS zero spacer length), is advisable.

Furthermore, we propose (or speculate) that the CLMS approach, though, not previously carried out, could be merged with novel interactomics techniques (proximity-dependent labeling by BioID or TurboID). Merging these two methods may bring spatial resolution to CLMS at a sub organelle level, because the BioID radius is estimated ~10 nm, and since CLMS mainly focuses on the proteins in the neighborhood of a given bait. These CLMS and proximal interactomics methods can be iteratively performed in living cells, i.e., the generated samples are the proximal species of a protein of interest labeled with biotin first and then cross-linked with interactors. Subsequently, perform streptavidin-biotin capture, followed by the digestion and cross-linked peptides identification of proximal interactors.

Besides the outstanding practice of CLMS in cross-linked peptide identification, it is worth noting that the failure to detect cross-links or only a few cross-links is related to several factors. For example, the lengths of the cross-linked peptides are too short or long, defective fragmentation of the cross-linked peptide, the cross-linked peptide is below the MS detection threshold, or the cross-linker is too short to link the appropriate residues. A few additional factors are: the sequence of interest is not fitting well with the cross-linker chemistry concerning reactive or cleavable residues, imperfect reaction conditions, the whole amount is too low, and extraction difficulties of the cross-linked peptides from a gel (in such a case,’in-solution’ digest is an alternative option). Therefore, the functional groups in a linker have to be tested and characterized thoroughly, as well as demonstrated precisely within the CLMS framework. Additionally, we addressed different protocols that are useful for digestion in CLMS methodologies, such as ‘in-gel’, ‘out-gel’, ‘in solution’, and FASP, with their successful usage. Alongside the CLMS application for identifying the protein–protein interactions, this review covers or describes the cross-linking methods for studying protein–RNA as well as protein–DNA interactions, which apparently arises as fertile ground for future CLMS utilization.

## Figures and Tables

**Table 1:** Few examples of a diverse set of chemical cross-linkers supported by different packages or tools [105,106,107,108,109,110,111,112].

Tools	Chemical Cross-Linkers Supported	Website
MeroX (StavroX included) [106]	BS2G, BS3/DSS, BS3/DSS-D0/D12, CDI, DC4, DSAU, DSBU, DSSO, DST, EDC, Formaldehyde(12), Formaldehyde(24), SDA	http://www.stavrox.com/Download_MeroX_Win.htm (accessed on 23 February 2021)
Spectrum Identification Machine for Cross-Linked Peptides (SIM-XL) [107]	DSS, DSG, DSSeb, DSS/DSG/DSSeb (with reporter ions only), XPlex C6N2, XPlex C3N2, XPlex C6Ac2, XPlex C3Ac2, Disulphide, zero-length	http://patternlabforproteomics.org/sim-xl/ (accessed on 23 February 2021)
Xilmass [108]	DSS (d0/d12), BS3(d0/d4), EDC and GA	http://compomics.github.io/projects/xilmass.html (accessed on 23 February 2021)
xQuest/xProphet [109]	BS3, DSS etc.	http://proteomics.ethz.ch/cgi-bin/xquest2_cgi/download.cgi (accessed on 23 February 2021)
XiSEARCH [110]	BS2G, SDA, BS3, DSSO, EDC, NonCovalent, Linear Search	https://www.rappsilberlab.org/software/xisearch/ (accessed on 23 February 2021)
Kojak [111]	BS3, DSS etc.	http://www.kojak-ms.org/param/cross_link.html (accessed on 23 February 2021)
pLink 2 [112]	BS2G, BS2G_heavy, BS3_heavy, DSS, EDC-DE	http://pfind.ict.ac.cn/software/pLink/ (accessed on 23 February 2021)

In addition to tools presented in this table other programs such as; XLSearch, ECL 2.0, MaxQuant, xTract, Protein Prospector, pQuant, mMass, CLMSVault, xVis, XlinkX, Mango, CLPM, Crux, DXMSMS, FINDX etc., can also be used for identification of cross-linked peptides [11,80,105].

## Data Availability

Data is contained within the article.
